# Gilles de la Tourette's syndrome in a patient with 47(XXX) syndrome: a case report

**DOI:** 10.1186/1752-1947-5-542

**Published:** 2011-11-05

**Authors:** Matteo Chiappedi, Silvia de Vincenzi, Roberta Dolci, Sara De Luca, Maurizio Bejor

**Affiliations:** 1 Don C Gnocchi Foundation, Piazzale Morandi 6, 2012 Milan, Italy

## Abstract

**Introduction:**

To the best of our knowledge, this is the first report of a comorbidity between Gilles de la Tourette's syndrome and 47 (XXX) syndrome. The clinical picture of Gilles de la Tourette's Syndrome is well described, while 47 (XXX) syndrome is much more rare and has a broader spectrum of possible phenotypic presentations.

**Case presentation:**

An Italian Caucasian girl was referred at the age of 11 to our Rehabilitation Center for anxiety and learning difficulties. The girl had already been diagnosed as having 47(XXX) syndrome; she had some rather typical features of the chromosomal abnormality, but she also showed a high level of anxiety and the presence of motor and vocal tics. When an accurate history was taken, a diagnosis of Gilles de la Tourette's Syndrome emerged.

**Conclusions:**

The possible interaction between peculiar features of these two syndromes in terms of neuropsychological and affective functioning is both interesting for the specific case and to hypothesize models of rehabilitation for patients with one or both syndromes. Executive functions are specifically reduced in both syndromes, therefore it might be hard to discriminate the contribution of each one to the general impairment; the same applies to anxiety. Moreover, mental retardation (with a significantly lower verbal cognitive functioning) poses relevant problems when suggesting cognitive behavioral or psychoeducational rehabilitative approaches.

## Introduction

47(XXX) syndrome, also known as triple X syndrome, was first described in 1959 by Jacobs and coworkers in a woman with ovarian failure [1]. The 47(XXX) karyotype has a frequency of one in 1000 female newborns, but this syndrome is not usually suspected at birth or childhood and is often diagnosed incidentally with prenatal diagnosis or following medical testing for infertility. Diagnosis is confirmed by karyotype analysis and the most common cause is lack of disjunction during maternal meiosis [2]. Patients with 47(XXX) syndrome do not usually present with major malformations, but rather subtle and highly variable clinical features such as high stature, poor motor coordination, language delay, and learning disabilities (often mild) [3]. In some cases patients may present some behavioral problems, such as hyperactivity, poor social interaction, depressive traits or mild depression: even though these psychopathological aspects seem to become less relevant once they leave school, there is an increase in prevalence of psychotic disorders in these patients during adulthood [4]. The intelligence quotient (IQ) of patients with 47(XXX) is thought to be on average 20 points lower than controls, with a significant discrepancy between the performance IQ and the verbal IQ (the latter being usually lower). As already mentioned, during adulthood these patients often present with premature ovarian failure and infertility.

Gilles de la Tourette's syndrome (TS) is a well known syndrome [5] that has a typical onset in childhood, mostly between five and six years of age [6]. It is defined by the occurrence of multiple motor and one or more vocal tics; even if not concurrent, they should be present almost every day, usually in bouts, for no less than 12 months (a 'free period' of less than three months is accepted). Tics should begin before the age of 18 and should not be a consequence of a substance or of a general illness [7]. Diagnosis is usually delayed more than five years from the start of symptoms, often because patients and their families tend to hide the symptoms [8]. Frequently associated symptoms include coprolalia, obsessive-compulsive disorder (or obsessive traits), attention deficit (with or without hyperactivity), and anxiety disorders.

In the present work, we report the case of a girl with prenatal diagnosis of 47(XXX) syndrome with mental retardation and symptoms of TS.

## Case presentation

The parents of an 11-year-old Italian Caucasian girl requested she be evaluated for a suspected anxiety disorder. Our patient had been found to have 47(XXX) syndrome after a prenatal investigation (amniocentesis due to the relatively high maternal age). She was born full term after a normal pregnancy; no problems were reported during the perinatal or the neonatal period. Her parents described her psychomotor development as normal. She went to kindergarten by the age of three without any significant difficulty. She attended primary school with no apparent problems for the first four years, and then learning difficulties increasingly began to be reported. Her parents also referred to a significant difficulty in making and maintaining friends. They suspected she may have a specific learning disorder, since a cousin of our patient was dyslexic.

When we first saw her she was attending the first year of the 'scuola secondaria di primo grado' (VIth grade); her parents described mainly difficulties in writing, a high level of performance anxiety and lack of self-confidence. They also mentioned the occurrence of occasional tic bouts, which they considered as a manifestation of anxiety.

She seemed at first to be scared by the clinical examination, and she felt embarrassed by her appearance, although she had no dysmorphic features and her auxologic parameters were in the normal ranges (height: 75th percentile, weight: 50th percentile). Results of her general physical examination were normal, with the exception of a partial dental malposition and signs of nail biting. Her neurological examination was almost normal as well, but multiple motor tics (simple and complex) involving her head, arms and mouth, and vocal tics, were evident. They tended to become almost continuous when she was asked to perform quick calculations or any other cognitive task subjectively considered difficult.

Although our patient considered tics to be 'not important' and refused to list involuntary movements she experienced, she admitted that they were 'annoying' in social situations. During the evaluation, the following tics were seen: eye blinking (both unilateral and bilateral), grimacing of the mouth, throwing back of the head, quick flexions or extension of one or both arms, knuckle popping, throat clearing, sniffling, and coughing. Most motor tics were partially masked by added voluntary movements to resemble 'normal', although always not fully appropriate, activities.

To better understand the reasons leading to her academic difficulties, a neuropsychological evaluation was proposed. She was very shy with therapists and doctors of either sex; her lack of self-confidence was evident and manifested as requests for supplementary explanations about the cognitive tasks she was asked to perform. The number of tics was noted to increase during evaluation, especially when she needed to find or adapt cognitive strategies on her own to successfully complete tasks. Details of the neuropsychological evaluation are given in Table 1; the tests are among those routinely used in Italy and recommended by the Italian Society of Child and Adolescent Neuropsychiatry (SINPIA). Spontaneous writing was not adequate for her school level in terms of orthography or in terms of text structure.

**Table 1:** Neuropsychological profile of our patient

Test/factor	Score/rating
WISC III:	

Total IQ	55

Verbal IQ	51

Performance IQ	70

Edinburgh Handedness Inventory	24/24 (right lateralization)

Constructional praxis	

Blockbuilding models	6/8 (slightly below average)

Borrelly-Oleron	3/8 (below average)

Modified Frostig's test	

QPVG	8th percentile

QPVMR	6th percentile

QIVM	13th percentile

Memory	

Digit span	Normal

Verbal span	Normal

Corsi Block Tapping Test	Z-score of -3

Tower of London (executive functions)	

Score	10th to 15th percentile

Decision time	Z-score of -0.5

Executing time	Z-score of -0.55

Total time	Z-score of -0.67

Violation of rules	6 (high)

Modified Bells' Cancellation Test	

Rapidity	25th to 50th percentile

Accuracy	25th percentile

Word reading	

Time	Z-score of -0.86

Correctness	15th percentile

Non-word reading	

Time	Z-score of -0.40

Correctness	>15th percentile

Text reading: rapidity/correctness	

Rapidity	Z-score of -1.6

Correctness	8 errors (average)

Text reading: comprehension	

Narrative text	7 correct answers out of 15 (below average)

Informative text	4 correct answers out of 15 (below average)

Writing	

List of words	15th percentile

List of non-words	10th percentile

Sentences (homophonic not homographs)	15th percentile

Dictation	60th percentile (slightly below average)

Mathematic abilities	

Numbers and calculation quotient	<50 (very poor)

Numbers quotient	<50 (very poor)

Calculation quotient	<50 (very poor)

WISC III = Wechsler Intelligence Scale for Children III.

IQ = Intelligence Quotient.

QPVG = General Visuo-Perceptive Quotient.

QPVMR = Reduced Motricity Visuo-Perceptive Quotient.

QIVM = Visuo-Motor Integration Quotient.

In short, our patient showed a mild mental retardation with a relatively higher performance level. Minor difficulties in reading and writing were noted, and her reading comprehension was below average. Her mathematical abilities were severely compromised.

Our patient showed an important deficit of planning strategies, including the visual-constructional praxis, visuomotor integration and short-term spatial memory. The tendency to act without considering rules (shown by her performance in the Tower of London test) can be read as an alteration in inhibitory function, which is a rather typical feature of TS [9]. Impairment in visual working memory tasks has been reported in patients with TS as well, but our findings do not allow us to exclude the effect of comorbidities [9].

## Discussion

A cognitive behavioral treatment was proposed (Habit Reversal Therapy [10]), but her parents refused consent to any intervention aimed at reducing her tics saying that 'they were not important'. Given their definite position, no pharmacological therapy was considered even if the high levels of anxiety shown by our patient would have required treatment. They accepted only an aide in order to allow their daughter to continue going to school (no differential classes exist in Italy; children with significant neuropsychiatric problems producing a condition of disability attend normal schools with a teacher and sometimes an educator to individually support them).

Our patient showed symptoms that can be produced independently by her chromosomal abnormality and by TS; namely, she has a significant impairment in the domain of executive functions (EFs). These can be defined as a group of neuropsychological abilities including action starting and control, flexibility, goal maintenance, and action planning [11]. They are thought to be important in those situations that involve planning, decision-making, error correction, and troubleshooting. They are also involved in the following scenarios: (a) unfamiliar situations, that is, where responses are not well learnt or contain novel sequences of actions; (b) dangerous or technically difficult situations; and (c) situations that require the overcoming of a strong habitual response or resisting temptation.

The frontal lobes are strongly involved in EFs [12] and a role for neural circuits involving the frontal lobes and EFs has also been advocated in TS [13]. A recent paper suggested that patients with TS exhibit significantly poorer performances in theory of mind (ToM) tasks: these tasks imply the ability to reason about mental states with a strong involvement of the frontal lobes. In patients with TS, ToM skills are thought to be reduced by a dysfunction in frontostriatal pathways involving ventromedial prefrontal cortex [14].

From a clinical and neuropsychological perspective, it is however hard to differentiate the specific contributions of the two disorders to our patient's impairment. Inhibition deficits are associated both with TS and with anxiety and with some forms of mental retardation; her verbal skills were reduced as expected in 47(XXX) syndrome, a fact that could have masked the reported deficit in verbal fluency tasks [14]. While the finding of a significant reduction of visuospatial memory (Corsi block-tapping test) is in line with reported deficits in visual or spatial working memory tasks [9], the finding of a normal verbal short-term memory seems to confirm that patients with TS do not show deficits in this subtype of working memory [15].

Another feature which is reported both in 47(XXX) syndrome and as a comorbidity of TS is anxiety; however, our patient showed a pattern of social anxiety which is thought to be typical of 47(XXX) syndrome, that is, her anxious reactions were triggered by social stimuli such as the need to meet new people [4].

Treatment for a patient with both these conditions can be challenging: behavioral interventions have usually only been applied to patients with a normal or near-normal IQ, as they require the patient to understand and apply strategies in order to reduce the frequency and duration of tic bouts. Moreover, the typical 'stickiness' of thought processes in individuals with mental retardation can reduce the efficacy of psychological interventions [16]. However, most drugs used to reduce the severity of tics have been poorly tested or are not recommended in patients with mental retardation (as they can worsen cognitive performance) [10].

The phenotypic expression of 47(XXX) syndrome in our patient could be described as 'intermediate': she did not present with malformative signs but had mental retardation (with the typical pattern of a significantly lower verbal IQ).

In summary, this case report shows a typical example of denial of the importance of a symptom (tic bouts) that was a source of impairment for our patient, together with her social anxiety, dismissed as 'not important' by the parents and by the girl herself. This lack of consciousness of the importance of a medical condition or of a symptom is a factor commonly seen in parents refusing rehabilitative treatment [17].

## Conclusions

To the best of our knowledge, this is the first report of a patient with 47(XXX) syndrome and TS. Although this kind of association is probably rare, a thoroughly evaluation of these patients could be of help in improving our understanding of these disorders but also in planning better tailored therapies and/or rehabilitative treatments. This is especially important because these patients are likely to experience the difficulties described in patients with either isolated 47(XXX) syndrome [4] and in those with isolated TS [18] in real-life situations.

## Consent

Written informed consent was obtained from the patient's next-of-kin for publication of this case report. A copy of the written consent is available for review by the Editor-in-Chief of this journal.

## Competing interests

The authors declare that they have no competing interests.

## Authors' contributions

MC and MC performed neuropsychiatric and general medical evaluation of our patient. SDV, RD and SDL performed the neuropsychological evaluation of our patient. All authors contributed to writing the manuscript, which they all read and approved in its final version.
